# Led into Temptation? Rewarding Brand Logos Bias the Neural Encoding of Incidental Economic Decisions

**DOI:** 10.1371/journal.pone.0034155

**Published:** 2012-03-30

**Authors:** Carsten Murawski, Philip G. Harris, Stefan Bode, Juan F. Domínguez D., Gary F. Egan

**Affiliations:** 1 Department of Finance, The University of Melbourne, Parkville, Victoria, Australia; 2 Department of Management and Marketing, The University of Melbourne, Parkville, Victoria, Australia; 3 Psychological Sciences, The University of Melbourne, Parkville, Victoria, Australia; 4 Florey Neuroscience Institutes, The University of Melbourne, Parkville, Victoria, Australia; 5 Centre for Neuroscience, The University of Melbourne, Parkville, Victoria, Australia; 6 Monash Biomedical Imaging and School of Psychology and Psychiatry, Monash University, Clayton, Victoria, Australia; University of Maryland, College Park, United States of America

## Abstract

Human decision-making is driven by subjective values assigned to alternative choice options. These valuations are based on reward cues. It is unknown, however, whether complex reward cues, such as brand logos, may bias the neural encoding of subjective value in unrelated decisions. In this functional magnetic resonance imaging (fMRI) study, we subliminally presented brand logos preceding intertemporal choices. We demonstrated that priming biased participants' preferences towards more immediate rewards in the subsequent temporal discounting task. This was associated with modulations of the neural encoding of subjective values of choice options in a network of brain regions, including but not restricted to medial prefrontal cortex. Our findings demonstrate the general susceptibility of the human decision making system to apparently incidental contextual information. We conclude that the brain incorporates seemingly unrelated value information that modifies decision making outside the decision-maker's awareness.

## Introduction

Every time we go shopping, we are confronted with a huge number of products to choose from. The modern consumer's conundrum is the impact on the human reward system of the plethora of brand logos that are aimed at influencing purchasing decisions. Of fundamental importance is to understand how reward cues associated with a brand can influence the neural encoding of subjective values in reward- and decision-related regions of the human brain. Understanding the effect of brands as complex cues that are designed to influence our decision-making is an increasingly important topic in the cognitive neuroscience of economic behavior. A number of models have been proposed to explain how brands impact on consumer behavior, e.g. by changing attitudes or emotions [1]–[3]. However, the mechanisms of branding still remain poorly understood [4]. In particular, it is unclear whether the effect of brands is limited to product-related purchase decisions or whether it can generalize to other incidental economic decisions. The latter would be expected if strong brands acted as reward cues and stimulated the desire for more reward. Preliminary support for this hypothesis comes from neuroimaging studies, which have shown that reward cues, including culturally salient brands, activate brain structures linked to reward processing [5]–[9]. The question remains though whether brand logos can also change the encoding of reward values in the brain for incidental economic decisions.

The reward value of different choice options is rarely based on reward size alone but instead reflects other attributes, such as the probability of receiving the reward, the delay before the reward is available, as well as the individual's attitudes, motivational state, and past experiences [10], [11]. A common task to study the encoding of subjective values of rewards in the brain is temporal discounting in which participants are asked to choose between a smaller, sooner reward and a larger, later reward [12]–[17]. Rather than always choosing the (objectively higher) delayed reward, participants trade off reward size and delay [18], [19]. By varying reward sizes of the choice options, the task elicits individual *indecision points* at which the smaller, sooner reward and the larger, later reward are equally likely to be chosen. Thus, the indecision point characterizes individuals' intertemporal preferences. It also provides a measure of susceptibility to brand influence if priming with a brand leads to a shift in preferences. Temporal discounting therefore constitutes a well-suited task to study the influence of brands on incidental economic decisions. Neuroimaging studies using temporal discounting tasks have demonstrated that the subjective value of choice options is encoded in a brain network including medial prefrontal cortex (mPFC), posterior parietal cortex (PPC), anterior and posterior cingulate cortex (ACC, PCC) and the ventral striatum (VS) [12], [14]–[16], [20]. Thus, these regions were the most likely candidates for an influence of brand cues on the neural encoding of subjective values.

Several studies have found that reward cues can directly influence goal pursuit, even in the absence of conscious processing [21]–[23], and that they can bias intertemporal preferences towards immediate reward options over those available in the future [24], [25] and vice versa [17]. Additionally, subliminally presented brand logos can bias valuations and choices, even when the decisions are unrelated to the cue itself [26]–[29]. Thus, subliminal priming offers a promising way to investigate the effect of brands without making the objective of the study obvious to the participants which, in turn, could provoke undesired reflective or opposing behavior.

**Figure 1:**
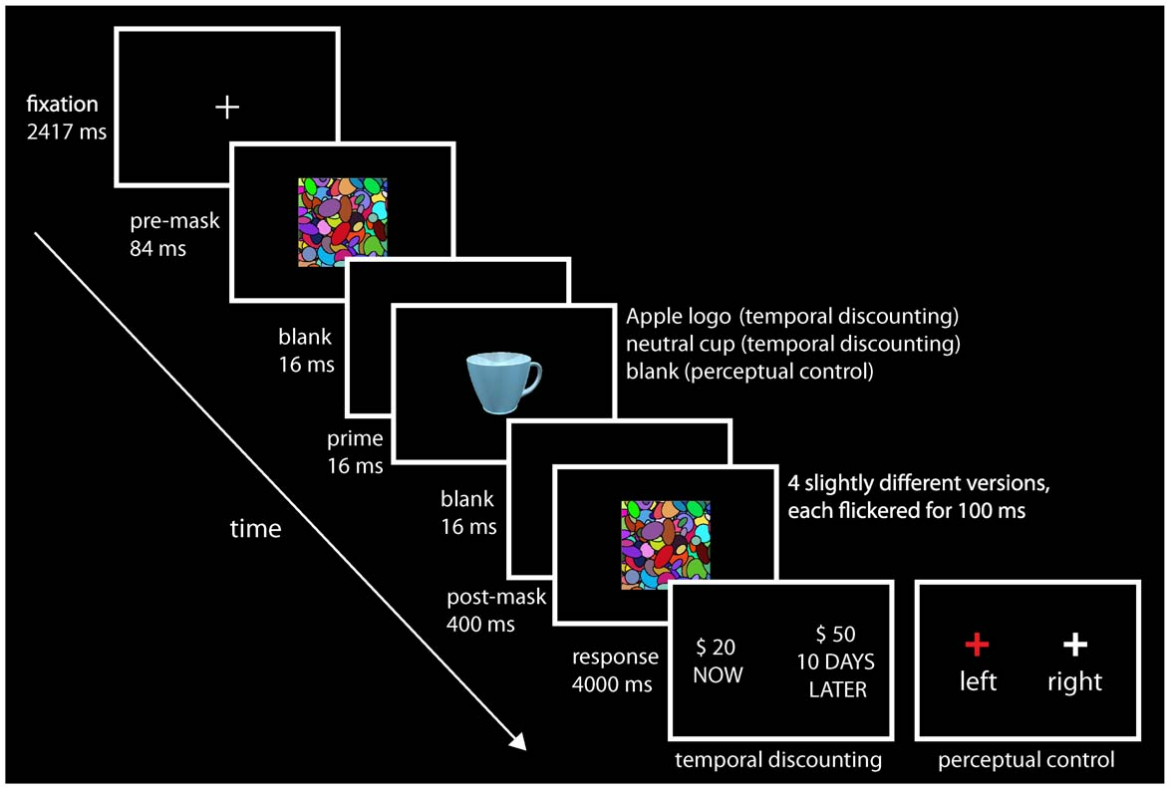
Stimuli and experimental paradigm. In each temporal discounting (TD) trial, a prime stimulus (Apple logo or neutral cup) was presented for 16 ms, flanked by two masks. The pre-mask was shown for 84 ms followed by a blank screen for 16 ms. The post-mask was shown for 400 ms, consisting of 4 slightly different versions, flickered for 100 ms each. The post-mask was further preceded by a blank screen for 16 ms, used to achieve optimal masking. During perceptual control (PC) trials, the prime stimulus was replaced by an additional blank screen (16 ms). Participants had to choose between two choice alternatives, presented randomly on either side of the screen. They indicated their choice by pressing a response button with their left or right thumb. For TD decisions, they had to choose between $20 now and a higher amount of money at some delay (shown on left side in the figure). For PC decisions, participants were asked to decide on which side the red cross appeared (shown on right side). The response period lasted 4000 ms.

In this functional magnetic resonance imaging (fMRI) study, we investigated the neural basis of the unconscious influence of rewarding brand logos by subliminally presenting either an image of the corporate brand logo of Apple Inc. (reward cue) or an image of a cup (neutral cue) to participants before they made a temporal discounting decision [14], [15], [20] (Fig. 1; see Materials and Methods). We chose the Apple logo for this study because according to market research, the Apple brand has been one of the most valuable consumer brands (http://www.millwardbrown.com/BrandZ). Furthermore, the Apple logo has been shown to create strong consumer-brand relationships and shape consumer behavior [2], suggesting the potential for a priming effect. Additionally, we obtained a priming effect with this stimulus in a separate study (unpublished data). We aimed to determine whether the brand logos would bias decision outcomes, even though the brand logo's domain was not related to the decisions. In line with other studies using strong reward cues [24], we hypothesised that priming with the brand logo would increase impulsive decision-making and lead to steeper discounting of future rewards.

Moreover, we aimed to identify brain regions in which the encoding of subjective value was modulated by the unrelated subliminal reward cue. In particular, we hypothesized that if brands impacted on the state of the reward system in general, our reward cues would influence the neural encoding of subjective values in a broad range of reward- and decision-related regions, e.g. medial prefrontal cortex, the striatum, anterior and posterior cingulate cortex and posterior parietal cortex. If brands unfolded their effect only during later stages of the decision process, an influence on the encoding of subjective value computation would be restricted to areas that explicitly encoded decision outcomes. In order to determine which areas were directly decision-related, we additionally used multivariate pattern classification methods [30]–[33] to search for brain regions that encoded decision outcomes.

## Results

### Brand relationship, stimulus awareness, and responsiveness

**Figure 2:**
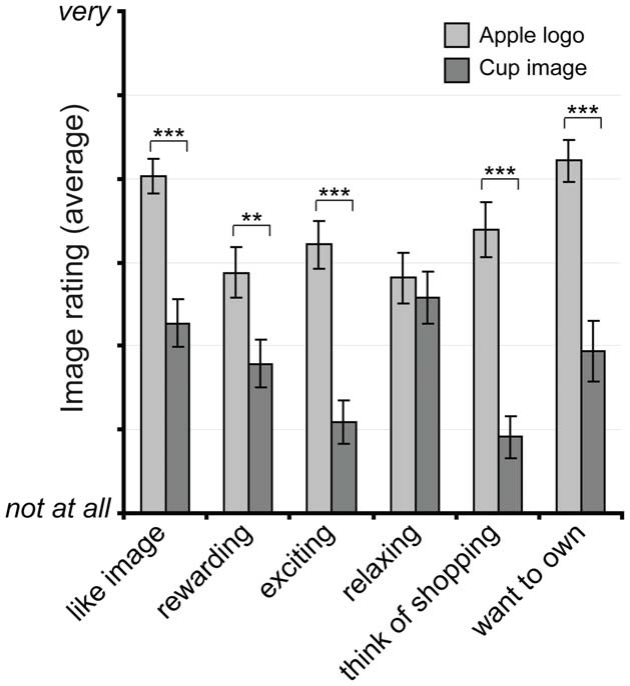
Evaluation of prime. An independent sample of 33 subjects (10 female; *M*_Age_ = 22.4; *range*: 20–25) were presented with images of the Apple logo and the cup as used in the fMRI study. They rated several statements about the images from 1 (Not at all) to 7 (Very). Like = “How much do you like this image?”; Rewarding = “How rewarding do you find the image above?”; Exciting = “How exciting do you find the image above?”; Relaxing = “How relaxing do you find this image?”; Shopping = “Does the image above make you think of shopping?”; Like = “How much would you like an Apple product/the cup?”. Displayed are average responses and standard errors.

The brand attitude survey administered after the scanning session showed that participants had a positive relationship with the Apple brand. The responses were (on a 7-point rating scale from 1 = “not at all” to 7 = “very”): “When I think of electronic products such as MP3 players, mobile phones, or computers, this brand is one of the first brands that comes to mind: [Apple logo]” (*M*_Salience_ ± *SE* = 5.61±0.24); “I love this brand: [Apple logo]” (*M*_Love_ = 4.92±0.29); “I would like to own one or more products of this brand: [Apple logo]” (*M*_Desire_ = 5.62±0.37); “I plan to buy one or more products of this brand in the next 6 months: [Apple logo]” (*M*_Intent_ = 3.92±0.42) (for individual results see Table S1). To investigate whether the Apple logo was also perceived as more rewarding than the supposedly neutral cup prime, we conducted a control experiment with an independent sample of 33 subjects (10 female, *M*_Age_ = 22.4, *range*: 20–25). Subjects were presented with an image of the Apple logo and the cup (as used in the fMRI study) and had to rate statements about these images on a 7-point scale (from 1 = “not at all” to 7 = “very”; for full list of statements see caption of Fig. 2). The results confirmed that the Apple logo was indeed considered more rewarding than the cup (*t*(32) = 3.17, *p*<0.01), was liked more (*t*(32) = 5.09, *p*<0.001), and was considered more exciting (*t*(32) = 5.36, *p*<0.001). The Apple logo also made subjects think of shopping more than the cup (*t*(32) = 6.50, *p*<0.001), and participants wanted Apple products more than the cup (*t*(32) = 4.59, *p*<0.001) (see Fig. 2). We further confirmed with two independent visibility tests that the primes were presented subliminally. In both tests, only one of the participant's 95%-confidence interval of *d′* did not contain zero, an indication that their performance was not significantly different from chance. Additionally, no participant's performance was different from chance in both tests, making it unlikely that primes were visible (see Table S2).

Participants chose the immediate reward in 43.9% of all trials (*SD* = 14.2), accompanied by overall balanced motor responses (*M*_Left_ = 48.8%). Temporal discounting decisions were significantly slower than perceptual control decisions on average (*M*_RT TD_ ± *SD* = 1564±287 ms; *M*_RT PC_ = 690±157 ms; *t*(15) = 10.68; *p*<0.001). Easy decisions were significantly faster than difficult decisions (*M*_RT Easy_ = 1,513±293 ms; *M*_RT Difficult_ = 1,615±292 ms; *t*(15) = 3.66, *p*<0.01). No differences in RT were found between Apple trials (*M*_RT Apple_ = 1,562±285 ms) and neutral cup trials (*M*_RT Cup_ = 1,562±292 ms; *t*(15) = −0.19, *p* = ns).

### Discounting behavior and priming

**Figure 3:**
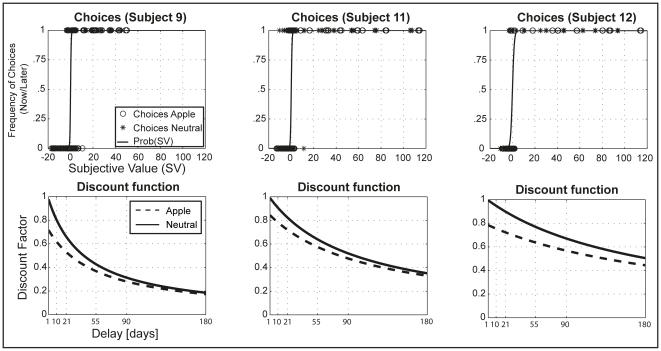
Behavioural results. The panels in the top row show actual choices by condition (Apple/neutral) and the fitted logistic functions for three example participants. The panels in the bottom row show participants' discount functions by condition (Apple/neutral), displaying the behavioral priming effect.

Participants' average discount rates *k* were computed by fitting the model in equation (I) (*M_k_* = 0.024±0.018). We investigated whether participants discounted delayed rewards differently when primed with the Apple logo by fitting a model which considers a prime-related ‘premium’ (*a*) in the discount factor (equation III). Nine (out of 13) participants displayed a positive ‘premium’, that is, they discounted delayed rewards more when primed with the Apple logo compared to the neutral condition (*M_a_* = 0.06±0.31) (see Fig. 3). On average, participants needed to receive $1.17 more for the ‘later’ option (at a delay of 180 days) in order to be indifferent between immediate and delayed rewards. The priming model fit the data better than the standard discounting model, as confirmed by likelihood ratio tests at individual level (*p*<0.05 for nine participants; comparisons of AIC/BIC confirm the better fit of the priming model; see Table S3 for parameter estimates and for model comparison results), and there was a significant priming effect as captured by parameter *a* in Equation (III) both, at individual level (*p*<0.05 for nine participants, see Table S3) and at group level (*p*<0.001, see Table S4). We did not find any significant correlation between the priming effect and participants' BIQ scores (reported in Table S1).

### Priming-related modulation of brain activation

**Figure 4:**
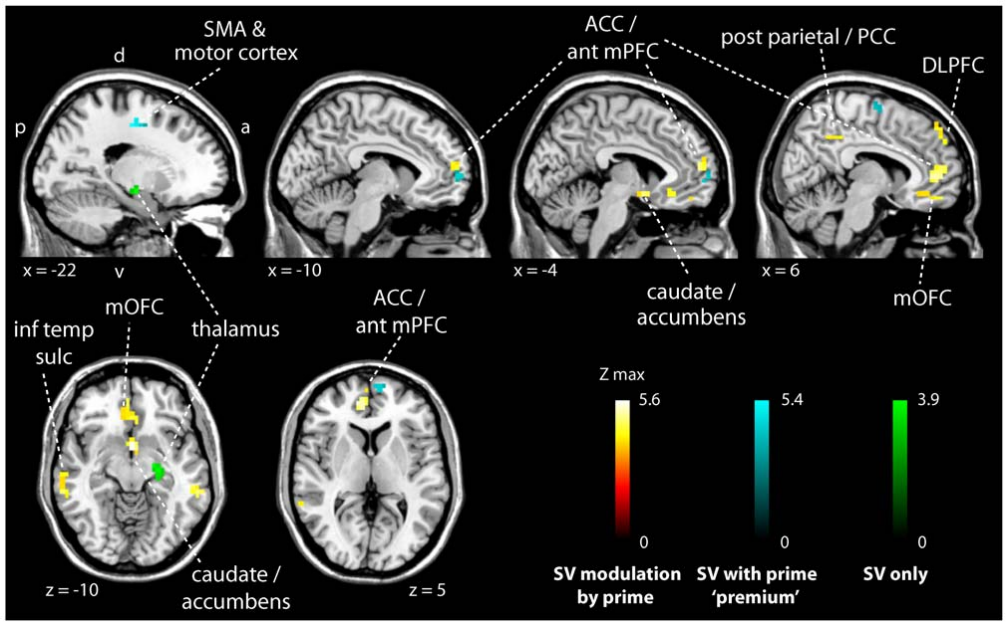
Regions in which activation for temporal discounting decisions was parametrically modulated by subjective value (SV). Using two parametric orthogonal regressors for SV and for the trial-by-trial interaction of priming and SV (see Materials and Methods), several regions were identified displaying the interaction, independent from general SV (‘*priming interaction model*’; displayed in yellow). These were anterior mPFC/ACC, mOFC, left PCC, the caudate nucleus/nucleus accumbens, inferior temporal sulcus, the IPS (not displayed) and medial occipito-temporal sulcus (not displayed). The only region, which encoded only SV but was not modulated by priming was the left thalamus (displayed in green). Regions displaying specific Apple prime modulation (‘*priming premium model*’; see Materials and Methods) of SV encoding were anterior mPFC, bilateral premotor cortex, and motor cortices (displayed in cyan). For MNI coordinates and statistics refer to main text.

Given that priming impacted on choice behavior, our main aim was to identify brain regions that were modulated by the priming effect on SV. First, we conceptualized the priming effect as a parametric interaction of SV and priming, independent of the general parametric effect of SV (‘*priming interaction model*’, see Materials and Methods). We found that this interaction effect modulated activation in a bilateral network containing anterior medial prefrontal cortex (mPFC) extending to frontopolar cortex (FPC, *Z*_max_ = 4.83, MNI -12 48 4), medial orbito-frontal cortex (mOFC; *Z*_max_ = 4.76, MNI -4 48 -16), the intraparietal sulcus (IPS, *Z*_max_ = 4.64, MNI -40 -56 32; *Z*_max_ = 3.66, MNI 36 -52 28), posterior cingulate cortex (PPC, *Z*_max_ = 4.79, MNI -12 -52 40), caudate nucleus/nucleus accumbens (*Z*_max_ = 5.02, MNI 0 8 -12), inferior temporal sulcus (ITS, *Z*_max_ = 4.72, MNI -64 -44 -4; *Z*_max_ = 5.58, MNI 60 -36 -12), and medial occipito-temporal sulcus (mOTS, *Z*_max_ = 4.11, MNI -20 -36 -16; *Z*_max_ = 4.24, MNI 24 -32 -16) (see Fig. 4). When the interaction between priming and SV was factored out, only a small cluster in the thalamus (*Z*_max_ = 3.88, MNI 20, -12, -12) showed parametric activation for prime-independent SV (Fig. 4). Thus, most regions that encoded SV were modulated by the priming effect.

Second, we modeled a parametric priming ‘premium’ (Π) for Apple trials only while treating the neutral cup prime trials as a baseline (‘*priming premium model*’, see Materials and Methods). This priming effect was found to modulate activation in mPFC (*Z*_max_ = 4.55, MNI 8 60 4), bilateral supplementary motor areas (SMA, *Z*_max_ = 4.22, MNI -8 -8 68; *Z*_max_ = 4.07, MNI 60 -20 44), and motor cortices (*Z*_max_ = 4.07, MNI -56 -16 32; *Z*_max_ = 5.37, MNI 20 -16 48) (Fig. 4). This could also be replicated when only correctly predicted trials (based on individually estimated discount rates) were used. Results are reported for a statistical threshold of *p*<0.05 (FWE corrected for clusters).

In the non-parametric whole-brain analyses, no brain region was found to directly differ in activation between prime conditions. Additionally, we used all 16 regions, which showed a parametric effect for SV in any of the priming models reported above, as regions of interests (ROIs; again using *p*<0.05, FWE corrected cluster threshold). We repeated the non-parametric control analyses for these ROIs. Again, none of these regions showed significant differences between priming conditions (*p*>0.05, corrected for multiple comparisons). This suggests that the priming effect was more accurately captured by the ‘*priming interaction model*’ and the ‘*priming premium model*’. All regions involved in the temporal discounting task, as revealed by the univariate control analyses, can be found in Table S5.

### Decoding of primes and decision outcomes

**Figure 5:**
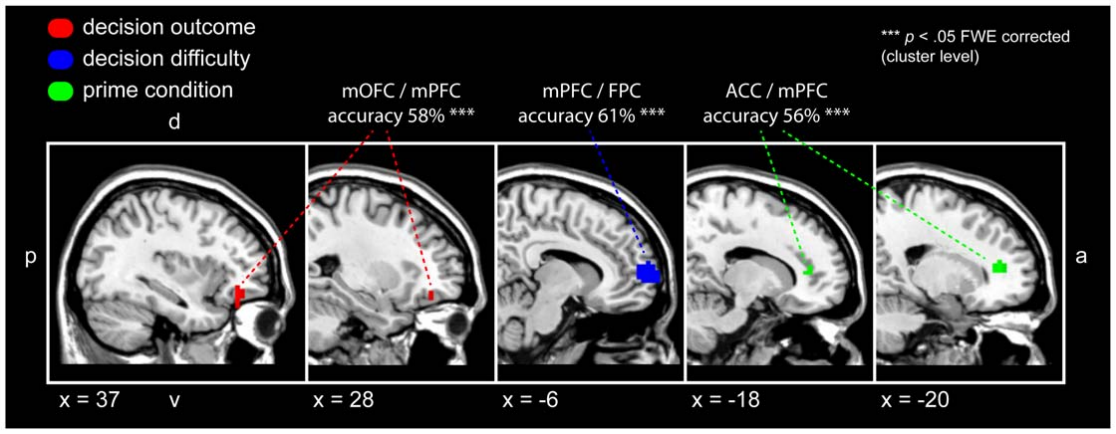
Decoding decision aspects and prime conditions. A moving searchlight decoder with a radius of 3 voxels was used to predict decision aspects from local brain activation patterns (*p*<0.05 FWE corrected cluster threshold for all). Clusters in anterior cingulate cortex (ACC)/medial prefrontal cortex (mPFC) (peak accuracy 58%) and visual cortex (peak accuracy 58%, not displayed) encoded the prime condition (Apple vs. neutral cup) for temporal discounting (green). A region in left mPFC/FPC encoded decision difficulty (‘easy’ vs. ‘hard’; peak accuracy 61%; blue). There was no difference between the Apple condition and the neutral cup condition (*p*>0.1). The decision outcome (‘now’ vs. ‘later’) could be decoded from right mOFC (peak accuracy 58%; red) for all TD decisions. More detailed prime-specific analyses could not be performed because seven subjects were too unbalanced in their binary decision outcomes (see main text). Error bars = SEM.

We also used a ‘searchlight’ variant of multivariate pattern classification [32] to decode decision components from spatial activation patterns (*p*<0.05 FWE corrected cluster threshold). Left anterior mPFC/ACC (accuracy 56%, *SE* = 0.17, MNI -20 40 12) and visual cortex (accuracy 58%, *SE* = 0.49, MNI -16 -99 8) encoded the specific prime condition (Apple vs. neutral cup), confirming that subliminal prime information was present in high-level decision areas (Fig. 5). Decoding accuracies in other brain regions, such as the ventral striatum were comparable (accuracy 56%, *SE* = 0.86, MNI -1 8 -12) but did not exceed the strict statistical threshold. Decision *outcomes* (‘now’ vs. ‘later’) were found to be encoded in right anterior medial orbito-frontal cortex (mOFC; accuracy 58%, MNI 36 36 12; four participants were excluded from this particular analysis because of unbalanced decision outcomes) (Fig. 5). Using smaller ‘searchlight’ clusters (radius = 2 voxels) confirmed the results and additionally showed a significant cluster in the left insula (accuracy 55%, MNI -24 -4 16). Finally, decoding *decision difficulty* (‘easy’ vs. ‘hard’ decisions as defined by the distance to the individual indifference points, see Materials and Methods) revealed a cluster in left anterior mPFC (accuracy 61%, MNI -8 60 8), overlapping with the cluster being modulated by SV (Fig. 5). It encoded decision difficulty for both Apple primes (accuracy 57%, MNI -12 60 8) and neutral cup trials (accuracy 54%, MNI -12 60 8; two unbalanced subjects with ≥1 run with <4 trials per choice in both conditions were excluded for this analysis). For the neutral cup condition, right mPFC (accuracy 59%, MNI 8 56 32) additionally encoded decision difficulty (see Table S6); this, however, was simply due to general activation differences at single-voxel level between ‘easy’ and ‘hard’ trials for the neutral cup condition in this region (see Table S7). Note that if many voxels in a given region show strong activation differences between conditions, then the local patterns in this region necessarily differ as well and the advantage of multivariate analysis vanishes. All results could again be replicated using a searchlight radius of r = 2 voxels. Results are reported for a statistical threshold of *p*<0.05 (FWE corrected for clusters).

## Discussion

In the present study we have shown that subliminal priming with a corporate brand logo influenced subsequent unrelated choices in a temporal discounting task. We further showed that priming with the brand logo affected the encoding of reward values of choice options in anterior mPFC. Several additional brain regions, including the striatum, ACC, mOFC, and posterior parietal areas were further modulated by the interaction of priming and subjective value (SV) on a trial-by-trial basis. Furthermore, our analyses demonstrated that information about the prime stimuli, decision difficulty, and decision outcomes was encoded in mPFC and mOFC, suggesting an involvement in making the final decision. Thus, the effect of brand priming was evident but not restricted to medial prefrontal decision areas. Rather, brands acting as complex reward cues appear to influence a wide range of reward- and decision related brain regions.

Given a choice between a smaller, immediately available reward and a larger, delayed reward, the relative value of the immediately available reward is modulated by both delay and magnitude of the delayed reward, independent of whether the rewards are primary such as fruit juice, or secondary such as money [14]–[16], [34], [35]. Previous studies have identified brain regions that contribute to this type of intertemporal decision-making. Inferior prefrontal cortex (PFC), medial PFC, temporal-parietal cortex, and peri-splenial PCC have been suggested to be involved in task components such as memory retrieval, planning, and cognitive control [20]. Other regions including mPFC, OFC, the ventral striatum, anterior insula, and PCC have been found to be particularly sensitive to value [14], [36]–[38]. In line with these studies, we found neural correlates of SV in a set of regions including mPFC/ACC, mOFC, PCC, DLPFC, and striatum. At this time, we do not yet understand why an extended network of brain regions encodes value. One suggestion is that value representations in different neural populations may contribute to different mental processes, ranging from automatic value associations to flexible, goal-directed planning [11], [39]–[41] and that different brain regions may support separate stages in the decision making process [42]. Here we could show that wide-spread medial prefrontal regions encoded decision difficulty, extending to OFC, which encoded decision outcomes. This supports earlier findings that linked OFC to the integration of cognitive and emotional information [43] as well as the encoding of stimulus and reward values [14], [44]–[46]. Others have shown that decision outcomes in reward-based decisions [47] and purchasing decisions can be predicted from signals in mPFC [48]. These reports, together with our results, suggest a prominent role of medial prefrontal regions in forming a decision outcome based on value representations. Note, however, that our study was not designed to disentangle the temporal information flow during the decision process and therefore cannot prove that the final decision was formed in medial prefrontal cortex. This process could also have involved several other regions while mPFC only represented the final decision outcomes.

Our main aim was to investigate the neural correlates of the priming effect. We found that temporal discounting decisions could be manipulated by subliminally presenting an image of an unrelated brand logo, which in turn was accompanied by a systematic shift in the encoding of SV in the brain. Here we show that priming with a brand logo had an effect on decision-making even though the prime was unrelated to the decision task, to subjects' expectations [49], to strong needs and desires [50], and was presented subliminally. On the flipside, these features of the prime might have weakened the priming effect in comparison to other studies which, in turn, might explain why others find even stronger priming effects [50]. We identified neural correlates of a priming ‘premium’ on SV in mPFC. However, this model alone might not fully capture the priming effect and lead to the wrong conclusion that priming only affects encoding in high-level decision areas and subsequent motor regions, which are related to response execution. Importantly, nearly all regions which encoded SV were susceptible to priming, including mPFC, mOFC, caudate nucleus, and PCC [14], [20], [36], [51]. We also found that not all regions which showed a priming effect on the encoding of subjective value also directly encoded the priming condition. This could simply be a problem of statistical power that could be more severe for smaller brain regions. Another explanation, however, is that in these regions (including the ventral striatum) the priming effect was expressed only indirectly by the observed modulation of subjective value encoding by priming. Thus, both methods revealed complementary information [32] about temporal discounting decisions and do not contradict each other. The combination of parametric analysis, which is more sensitive to graded variables, and multivariate pattern classification analysis, which is more sensitive to distinct classes of variables, has been shown to be highly efficient for dissociating different neural mechanisms [52].

These findings support the assumption that priming affects the general state of the reward system by biasing how reward cues are initially perceived in a trial. Medial PFC might then integrate representations of decision values which in turn becomes the basis for choice [53]. Note that our results do not imply (and we do not conclude) that all regions we found to be affected by priming play the same role in subjective value processing. Given the poor temporal resolution of fMRI our study cannot distinguish between feed-forward and feed-back processing and tracking of the information flow through this network requires future studies. We do conclude, however, that the priming effect was not an ‘add-on’ that occurred only during the final stages of the decision process.

The precise neural mechanism behind the brand priming effect on discounting behaviour cannot be resolved with our study alone. One possibility is that the brand could be regarded as a simple conditioned stimulus that triggered goal-directed behavior (‘wanting’) [54]. Alternatively, the brand prime could have activated a general goal, or motivational state, related to the desire to purchase a product. Immediate monetary rewards (steeper discounting) would facilitate achievement of this goal [25], [36]. Such goals or motivational states do not rely on conscious representations [21], [55], [56]. Others have shown that tagging the delayed choice option with words reminding subjects of important events that will occur at the same time in their future decreased discounting [17]. This was explained by triggering future-oriented thinking via episodic memory activation and was accompanied by differential activation in anterior cingulate cortex (ACC), amygdala and dorsolateral prefrontal cortex in the tagged condition. The brand primes in our study might have activated episodic memories related to immediate rewards. Note, however, that other explanations, such as the involvement of emotions in brand processing, cannot be ruled out.

We suggest that the reward value attached to the subliminally presented brand logo systematically biased the value representation of choices in the decision network. In support of this view, it has been shown that not only novel and explicitly conditioned stimuli [57], [58] but also cultural objects, related to wealth and social status, can activate the reward circuit [5]. A brand logo can substitute for such objects, as reported in a study in which positive brands led to consumer attachment and strongly affected behavior [2]. The presentation of brand logos has been shown to engage reward-encoding brain regions, including ventral striatum and anterior mPFC [6], [8], [59]. In our study, presentation of a brand logo to participants with a strong positive attitude towards the Apple brand is likely to have activated the reward system, with immediately available reward alternatives being assigned a higher relative value. This hypothesis is also supported by our control study that showed strong associations between the Apple brand with reward and shopping. These value signals may have been projected to motor regions, which could explain the prime-related shift in value representation in motor regions in our study. Our data, however, does not allow us to determine whether this information purely serves response preparation or whether there was a more direct involvement of motor regions in the decision process itself.

One difficulty of interpretation in this study is that priming might have affected behavior in a number of ways, suggesting that the nature of prime-related brain processes may have been incompletely measured. We addressed this possibility by combining univariate parametric analysis with multivariate decoding analysis. Multivariate decoding can detect fine-grained information beyond the resolution of the voxel grid [30] and has previously been used to decode reward-based decisions [12], [44], [47], [48]. It allowed us to identify regions, which were directly linked to decision difficulty and decision outcomes. Please note that this analysis served the purpose of directly predicting these decision aspects from brain activity patterns, making use of the high sensitivity of multivariate decoding for information about distinct classes. The graded encoding of subjective values, however, was better captured by parametric univariate models. Thus, both methods revealed complementary information [32] about temporal discounting decisions and do not contradict each other. Additionally, we used several approaches, considering a prime-related ‘premium’ as well as an interaction between priming and subjective value, both directly derived from behavioral models. However, we acknowledge that the underlying discounting functions, and potentially also priming effects, may be reflected differently in brain activity when different tasks are used [12].

We have also assumed that the primes were processed outside of the participants' conscious awareness, as confirmed by detailed debriefing interviews and by the two post-experimental tests that simulated the viewing conditions in the fMRI experiment. Our visibility tests provide strong indication that participants did not consciously perceive the prime images. There is no unanimously agreed criterion to determine prime visibility though [60] and residual visibility on single trials can never be ruled out with absolute certainty. This manipulation allowed us to avoid participants thinking about the experimental manipulation during the experiment. Since in the present study prime images were unrelated to the temporal discounting task, in any case residual visibility would not challenge the effect of incidental brand primes on the encoding of value.

It is well known that subliminal primes can affect goal pursuit [21], attitudes [61] and behavior in many ways [22], [23], [62]. We further note that our participants did not dramatically change (or reverse) their decisions. Subliminal exposure to reward cues may be more likely to prime a short-lived state (or tendency) that would nudge decision-makers towards more immediate rewards. We suggest that this may be one way the brain subserves decision making in highly complex environments [43], [63].

Due to limitations imposed by fMRI, our study was restricted to the use of one brand logo only and future studies with larger sample sizes are needed, using a larger set of priming stimuli and control stimuli to test the generalisation as well as the precise mechanisms behind our results. Future studies should also compare to what extent priming stimuli themselves would activate reward-related brain regions when they are not followed by an economic decision. In addition, further research is required to investigate how the brain processes and resolves the dynamic multiplicity of reward cues that we are exposed to in every-day life, of which brand logos are just one example. Other studies are required to investigate how a symbol can become a reward cue and how cue value may be integrated with the reward values of choices in decision making.

The findings of our study suggest that the formation of economic preferences is more complex than traditional models suggest and is influenced by many factors other than core decision parameters. Arbitrary symbols, such as corporate brand logos, can act as strong reward cues and affect decisions outside their initial domain, and are associated with a systematic bias in the encoding of reward values in the brain.

## Materials and Methods

### Ethics statement

The study was approved by the Economics and Commerce Human Ethics Advisory Group of The University of Melbourne (Ethics ID 0830289) and was conducted according to the Declaration of Helsinki. All subjects gave informed written consent.

### Participants

Eighteen right-handed adults (8 female; *M*_Age_ = 22.9 years; *range* = 19–29; normal or corrected to normal visual acuity) took part in the fMRI study. All were selected based on a behavioral pre-test with a similar task, which was further used to calibrate the task for the fMRI session. Three participants were excluded because of technical problems with data recording. Another two participants' data were excluded because they almost always chose the delayed reward. This suggests that they did not make decisions on a trial-by-trial basis but adopted a default strategy, or that the task was not calibrated properly. Therefore, we could not fit our models to these participants' responses. The final sample contained 13 participants (6 female, *M*_Age_ = 21.2 years, *range* = 19–29).

### Tasks

#### Main task

Participants completed temporal discounting (TD) and perceptual control (PC) trials, presented in random order. In TD trials, one masked prime image preceded the presentation of a decision task. The decision involved the choice between an immediate reward (always ‘$20 today’) and a larger, later reward, which varied by amount and delay (all in Australian dollars). Participants were presented with six different delays (1, 10, 21, 55, 90, and 180 days) and six different amounts per delay (from $20.10 to $385.16), resulting in 36 combinations, each presented three times during the fMRI session in different runs. Delayed amounts were chosen individually (based on pre-tests) such that the expected number of choices of the immediate reward (‘now’) equaled the expected number of choices of the larger, later reward (‘later’). Two different types of primes were used. The first one was the Apple logo in three different colour versions, which are used by Apple Inc. in branding and advertising. These were randomly assigned to the ‘Apple prime trials’. The Apple logo was chosen based on the results of a behavioural study in which it showed a stronger priming effect than other logos as well as smiling faces [64]. The second prime was an image of a cup (‘cup prime trials’) drawn from a realistic object database (Michael J. Tarr, http://www.tarrlab.org/) and modified to match the Apple logo versions for color and size. The cup motif was selected as a neutral prime for its perceived familiarity, comparable visual complexity, and relative affective neutrality. In TD trials, each amount-delay combination was combined with each of the two prime stimuli, resulting in 216 trials in total. A mask displaying a dense arrangement of colored ellipses was used in order to render the primes invisible. In the PC task (108 trials) participants had to respond to the colour of one of two crosses, each presented on either side of the screen (Fig. 1).

Each trial began with the central presentation of a fixation cross (2417 ms), followed by a combination of a forward (84 ms) and a backward mask (400 ms) between which the prime image (16 ms, additionally flanked by blank screens for 16 ms, respectively) was shown. Finally, a response screen (RS) was shown (4000 ms), displaying two choice alternatives ($20 now vs. a larger, delayed reward) on either side of the screen. Participants indicated their choice by pressing either the left or right response button on a response box using their thumbs. Stimulus and response side combinations were pseudo-randomized for each trial with each stimulus appearing equally often in each functional run (using *OptSeq*; [65]). The dissociation of choice options and response buttons avoided confounds of decision outcomes and motor responses. For the PC task, a blank screen (16 ms) replaced the prime stimulus between the masks and participants were asked to indicate on which side the red cross appeared on the RS (the other cross was always white). The sequence was randomized for each functional run. In each run (duration 378 s) participants were presented with 54 trials (7000 ms each) controlled by Psychtoolbox for MATLAB 7.0 (The MathWorks, Inc.). Stimuli were presented via a projector (resolution 1024×768 pixel, 60 Hz) that was placed at the front-end of the scanner and projected onto a 100×75 cm screen placed in front of the scanner. Participants viewed the projection via a mirror fixed onto the head coil. The visual angle was 5.0° for all stimuli.

#### Brand and impulsiveness questionnaires

After the fMRI sessions, subjects completed a questionnaire to elicit their relationship with the Apple brand. Participants rated statements related to the Apple brand on a scale form 1 (strongly disagree) to 7 (strongly agree). The single scales were then added up and standardized into a ‘brand-score’. Additionally, participants completed the Barrett Impulsiveness Scale (BIS-11, items 1 to 18) using a set of statements that had to be rated on a scale scale from 1 (Rarely/Never) to 4 (Almost Always/Always) to assess general impulsiveness [66]. We administered this instrument to test whether susceptibility to priming was modulated by individual trait impulsivity.

#### Visibility tests

Subsequently, participants completed two visibility tests, using the same masking sequence that was used for the fMRI experiment. First, a simple detection task was used to test if participants were able to detect the presence of *any* stimulus at all when explicitly paying attention to the stimulation (which was not the case in the fMRI study). For this, a masked image (50% Apple logo, 50% neutral cup) was shown in half of the trials and no image was shown between the masks in the other half. Subjects were asked to respond ‘yes’ via button press if they saw an image in between the masks, and ‘no’ otherwise. The second task tested whether participants could identify any particular image. One of four possible masked images (Apple logo, the neutral cup, another corporate logo, and another household object) was shown in every trial, followed by a response screen showing all four images. Participants were asked to select the image they believed themselves to have seen. In Test 1, we counted a ‘yes’ response in a trial in which an image was presented as a hit, and a ‘yes’ response in a trial in which no image was presented as a false alarm. In Test 2, we counted the choice of the Apple logo in a trial in which the Apple logo was presented as a hit, and the choice of the Apple logo in a trial in which another image was presented as a false alarm. 95% confidence intervals for *d′* were bootstrapped by resampling responses at subject level from the empirical distributions and computing *d′* based on re-sampled responses (n = 1000).

#### Participant payments

Participants received a total of $50 for participating in both the screening session and the fMRI experiment. In order to make the task incentive-compatible, participants were given the chance to win one of the choices they made during each session by rolling a die. On rolling a six, one of their TD choices was drawn randomly and paid out. If the participants chose the immediate reward in that trial, they received $20 in cash. Otherwise, the reward was transferred into the participant's bank account after the given delay.

### Functional imaging

Functional MRI images were acquired at the Royal Children's Hospital (Melbourne, Australia) using a Siemens 3T Magnetom Trio MRI scanner (Erlangen, Germany) with a standard head coil. Foam padding around the head was used to reduce head motion. T2*-weighted functional images of the whole brain were collected using a gradient-echo, echo-planar imaging (EPI) sequence (TR = 2000 ms, TE = 35 ms, 32 transverse slices acquired in ascending interleaved order, 3.6×3.6×4 mm^3^ voxel size, 64×64 matrix in a 230 mm field-of-view). Data was acquired in six functional runs, separated by rest periods of 1 min. During each run, 189 functional images were recorded. The first two images were discarded to allow the MR signal to reach a steady state. Additionally, a high-resolution, T1-weighted anatomical image was collected for each participant at the beginning of the scanning session for co-registration (TR = 1900 ms, TE = 2.59 ms, flip angle = 9°, 92 sagittal slices, 0.8×0.8×0.9 mm^3^ voxel size, 338×338 matrix in a 270 mm field-of-view).

### Data analysis

#### Behavior

Previous studies have shown that most individuals discount future rewards hyperbolically, that is, the cost of waiting increases linearly in the delay [14], [67], [68]. Thus, a participant's subjective value *SV* of a reward *R* received in *D* days was given by
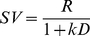
(I)where *k* is the participant's discount rate. Given the variability of participants' decisions we further assumed that decision errors have a logistic distribution and that the probability that a participant chose delayed reward *R* at delay *D* over an immediate reward of 20 was given by
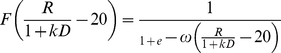
(II)where 1/ω is the variance of the logistic distribution. Participants' discount rates were estimated by fitting a logistic model to their choices [14], which was found to provide the best fit for our data.

In the first priming model (‘*priming premium model*’) we assumed that participants discounted delayed rewards *differently* when primed with the Apple logo. We assumed that the subjective value SV^*^ of reward *R* received at delay *D* was given by
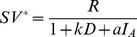
(III)where *a* is the ‘premium‘ on the discount rate due to the priming effect, and *I_A_* is a dummy variable for the Apple condition taking value 1 when the trial is an Apple trial and 0 otherwise. We estimated parameters *k*, *a*, and ω in equations (I) and (III) with maximum likelihood estimation at the subject level. The ‘premium’ on SV induced by priming (Π) was computed as the difference between the priming-induced subjective value (SV^*^) and the subjective value (*SV*) as follows:
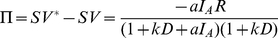
(IV)


In a second priming model, we defined the variable Σ as the interaction between the priming ‘premium’ (*a*) and the priming-induced subjective value (SV^*^), referred to as ‘*priming interaction model*’
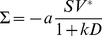
(V)This model tests the hypothesis that the priming effect increases with SV (i.e. is less pronounced at low levels of SV). Besides priming effects, we additionally analyzed decisions according to other parameters. First, we defined *decision difficulty* as a function of the distance Δ between the subjective value (SV) of the delayed reward, and the immediate amount where
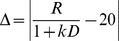
(VI)Difficulty of a choice is then defined as
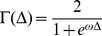
(VII)Γ is bounded by 0 and 1. It increases when the distance Δ between the SV of the delayed reward and 20 decreases, and is maximal when Δ is zero, that is, when the SV of the delayed reward is equal to 20 [69]. Parameters *k* and ω in Γ(Δ) were estimated in the maximum likelihood estimation of equation (I). A ‘hard’ decision was defined as a decision for which the difficulty index Γ was higher than the participant's median difficulty index.

#### Univariate fMRI analysis

For all analyses, functional images were first slice-timing corrected, realigned to the first functional image of the first run, co-registered to the individual T1-weighted anatomical images, normalized to the MNI template (as implemented in SPM2; http://www.fil.ion.ucl.ac.uk/spm/) and re-sampled to an isotropic spatial resolution of 4×4×4 mm^3^. Data were smoothed with a Gaussian kernel of 8 mm full width at half maximum (FWHM) to account for anatomical variability and to satisfy the assumption of Gaussian random field theory.

Our main analysis examined the influence of the primes on brain activation with respect to the encoding of subjective value (SV). We searched for brain regions in which the encoding of SV was either influenced by priming or independent from priming. For this, different general linear models (GLM) were estimated on an individual subject level that modelled each trial beginning with the visual presentation to the end of the response period (4500 ms). For the first model, Apple trials, cup trials, and control trials were modelled separately. We based our analysis directly on the ‘*priming interaction model*’ and estimated the trial-by-trial SV and the priming effect using equation V (see above) to incorporate an interaction between SV and the priming effect on the discount rate. This means, for each temporal discounting trial general SV was modeled parametrically and the interaction with the priming effect was included as a second, orthogonalized parametric regressor (control trials were modelled non-parametrically since these had no SV). General (prime-independent) SV was factored out in order to search for regions, which displayed *prime-specific* encoding of SV. In an additional analysis, we then reversed the order of parametric regressors and factored out the interaction with the priming effect in order to search for regions in which the encoding of SV was *not* influenced by priming.

As a second approach, we used a different model, based on the ‘*priming premium model*’ (equation III, see above) that assumed a specific priming ‘premium’ for the Apple logo primes. Apple trials, cup trials, and control trials were again modelled separately with SV incorporated as a parametric regressor (of no interest) and factored out for each TD trial. For Apple prime trials only, a parametric Apple-specific regressor was incorporated and orthogonalized to the SV regressor to test for modulation of brain activity specifically related to the Apple prime. A cluster significance threshold of *p*<0.05 (FWE corrected) was used for all group-level statistical analyses. Taken together, these analyses independently tested for neural correlates of both behavioural priming-models as well as for non-modulated SV encoding.

These analyses were further supported by several control analyses in which TD decisions were contrasted against baseline as well as against PC decisions. For this, a general linear model (GLM) was estimated on an individual subject level that modelled each trial as belonging to one of the two decision tasks (TD and PC). Furthermore, TD decision trials were modelled as belonging to Apple trials and cup prime trials, resulting in 3 boxcar regressors. The mean activation differences between prime conditions were based on analyses for the whole brain as well as for individual regions of interests (ROIs), which were constructed using the group-level baseline contrast for parametric modulation of general (prime-independent) SV encoding. In further control analyses, we additionally estimated models incorporating regressors for the decision outcomes (‘now’ vs. ‘later’) and decision difficulty (‘easy’ vs. ‘hard’) for TD trials within the two prime conditions separately.

#### Multivariate fMRI analysis

We additionally used multivariate pattern analyses (MVPA; [30], [33]) to predict the *prime condition*, decision *outcomes* and decision *difficulty* for TD trials. In order to search for predictive regions throughout the whole brain in an unbiased, non-circular fashion, we ran ‘searchlight’ decoding analyses [32].

The functional images were first pre-processed using slice-timing correction and motion correction. Further normalization and spatial smoothing was not performed in order to preserve as much of the original information in the data as possible [30]. For each analysis, a Finite Impulse Response (FIR) model was estimated (as implemented in SPM2), which requires few assumptions about the exact shape of the BOLD response and therefore preserves more information in the data [70]–[72]. A high-pass filter with a cut-off of 128 s removed low frequency drifts in the time series at each voxel. The model subdivided each trial into six distinct time bins of 2000 ms ( = 1 TR) covering a total of 12000 ms in order to capture all trial-related activation, taking into account the hemodynamic delay. The first time point (t = 0) was defined as the beginning of the image acquired at the start of the visual stimulation (4500 ms). For each time bin, the cortical response was estimated separately by one parameter (for details see [70]). For decoding of the prime condition, Apple and cup prime trials were modelled separately, resulting in 2 (type)×6 (time bins) = 12 regressors for each run and each subject. MVPAs were run on all *n* possible searchlight-clusters for all six time bins. A radius of r = 3 voxels was used to define spherical searchlight-clusters with *k* voxels (c_1…*k*_) surrounding each central voxel (v*_i_*_…*n*_) [48], [72]. The FIR-parameter estimates for the voxels from each cluster were extracted separately for both prime conditions at a given time bin for each run and each participant and then transformed into *pattern vectors* (for illustrations see [30], [70]). Starting with the first cluster around voxel v_i_, the pattern vectors from all but one functional run were used as a ‘training data-set’ and passed to a linear support vector machine (SVM) classifier [73] with a fixed regularisation parameter C = 1 (using LIBSVM 2.91, [74]). Based on the ‘training data-set’ the classifier estimated a classification hyperplane to separate patterns from both conditions. This hyperplane was then tested by classifying the pattern vectors from the independent, left-out functional run (‘test data-set’). Please note that the classifier operated on pattern vectors based on average regressors for each condition and not on single trials. This avoided potentially biasing the classification by small run-by-run variations in trial numbers per condition, as naturally occurs for decision tasks. It also means that there was always one vector for each condition with balanced training and test data-sets for each run and chance level was always 50% for two alternatives. The quality of the classification, denoted as the *decoding accuracy*, was calculated by averaging across six cross-validation steps, using each run as the independent ‘test data-set’ once. This procedure also controlled for over-fitting and false-positives [12], [48]. The classification analysis was then repeated for each cluster in the brain (separately for each time bin), resulting in a three-dimensional brain map of decoding accuracy values for each time bin, individually for each participant. These maps were then normalized to MNI space and smoothed with a Gaussian kernel of 8 mm FWHM and finally subjected to standard random-effects group-level statistical analyses as used by us and others [44], [48], [70]–[72], using a cluster significance threshold of *p*<0.05 (FWE corrected). Taking into account the delay in the hemodynamic response, only the last two time bins were statistically analysed because earlier time bins could not reflect trial-related activity [12]. Using a similar FIR model, we confirmed that no stimulation-related visual activity was found in earlier time bins (by means of univariate analysis) and that no decision-related information could be decoded from time bins 1 to 4. We also replicated all decoding analyses based on GLMs, which modelled the HRF instead of using FIR models [48]. The results entirely confirmed the reported findings (however, sometimes at lower significance thresholds) and did not reveal additional predictive regions; these results are not reported here.

In the same way, we decoded different aspects of participants' decisions from local spatial activation patterns. Three independent analyses were conducted to decode a) the *priming condition* (Apple vs. neutral cup), b) the *decision outcome*s for TD decisions (‘now’ vs. ‘later’) and c) the *decision difficulty* for TD decisions (‘easy’ vs. ‘hard’). The only difference between these analyses was that different regressors were estimated as the basis for subsequent decoding. In order to control for potential biases and smaller brain structures [48], we replicated all analyses using a searchlight radius of 2 voxels.

Finally, we conducted multivariate pattern classification analyses *within* prime conditions separately. Note that for the latter analyses with half of the trials per condition, decoding of *decision outcomes* could not be performed for prime conditions separately because of the small number of trials per condition.

## Supporting Information

Table S1 **Questionnaire responses.** Note: Sbj = subject. Ratings from 1 (strongly disagree) to 7 (strongly agree). Salience = “When I think of electronic products such as MP3 players, mobile phones, or computers, this brand is one of the first brands that comes to mind: [Apple logo]”; Love = “I love this brand: [Apple logo]”; Own = “I own one or more products of this brand: [Apple logo]”; Desire = “I would like to own one or more products of this brand: [Apple logo]”; Intent = “I plan to buy one or more products of this brand in the next 6 months: [Apple logo]”; BIQ = sum of BIS-11 Attention, Motor, and Self-Control factor scores.
(DOCX)

Table S2 **Results of visibility tests.** Note: The table shows d′ for the two visibility test as well as bootstrapped 95% confidence intervals of d′. In Test 1 (2AFC prime vs. control blank screens), a hit was defined as a ‘yes’ response in a trial in which a prime was displayed and a false alarm was defined as a ‘yes’ response in a trial in which no prime was displayed. In Test 2 (4AFC), a hit was defined as an ‘Apple’ response in a trial in which the Apple logo was displayed and a false alarm was defined as an ‘Apple’ response in a trial in which a different prime was displayed. Confidence intervals were bootstrapped at subject-level by resampling responses from the empirical distributions and computing d′ based on resampled responses (n = 1000). Two of the participants, marked ^#^ in the table above, did not have any hits in the Apple condition and d′ could not be computed.
(DOCX)

Table S3 **Comparison of baseline model and priming model.** (a) Parameter estimates. Note: Sbj = subject; k = discount rate; SE = standard error of parameter estimate; omega = reciprocal of the variance of logistic distribution; a = priming premium. For equations and details see Materials and Methods. (b) Model comparison. Note: Sbj = subject; B = baseline model (equation I); P = priming premium model (equation III); Δ = P – B; p(LR) = *p*-value of likelihood ratio test; AIC = Akaike Information Criterion; BIC = Bayesian Information Criterion.
(DOCX)

Table S4 **Mixed-effects model of priming effect.** Note: The table above shows estimates of fixed effects in a non-linear mixed effects model of participants' choices at group level based on Equation (III). Standard errors of coefficient estimates are given in brackets. ^***^ *p*<0.001; ^**^ *p*<0.01; ^*^ *p*<0.05.
(DOCX)

Table S5 **Baseline contrasts for univariate analyses.** Notes: Temp. Disc. = temporal discounting decisions; Control = perceptual control decisions; SV param = parametric effect for general subjective value (no priming effect accounted for); L = left; R = right; clusters determined by *Z* value>1.96 and a family-wise error (FWE) corrected cluster significance threshold of *p*<0.05; coordinates are given in MNI space. Please note that the perceptual control task also activated parts of the same network as the temporal discounting task and thus did not constitute an optimal task to isolate regions that were uniquely involved in discounting.
(DOCX)

Table S6 **Results decoding decision aspects.** Note: TD = temporal discounting decisions; L = left; R = right; M = mean; SEM = standard error of mean; apple = Apple prime condition; cup = neutral cup prime condition; mPFC = medial prefrontal cortex; ant = anterior; inf = inferior; DLPFC = dorsolateral prefrontal cortex; FPC = frontopolar cortex; temp = temporal; sulc = sulcus; gyr = gyrus; ‘#’ 2 subjects excluded because of unbalanced runs for both prime conditions (n = 11); all results *p*<0.05 family-wise error (FWE) corrected at cluster level; all analyses performed using unbiased searchlight decoding with radius r = 3 voxels. Coordinates are given for peak voxel in MNI space.
(DOCX)

Table S7 **Differential contrasts univariate analyses.** Note: TD = temporal discounting decisions; Cup = neutral cup prime condition; L = left; R = right; clusters determined by *Z* value>1.96 and a family-wise error (FWE) corrected cluster significance threshold of *p*<0.05; coordinates are given in MNI space. Other contrasts (hard>easy; cup>apple; apple>cup; apple-hard>apple-easy; apple-easy>apple-hard; cup-hard>cup-easy) did not reveal significant results. Interestingly, easy choices led to more activation in anterior mPFC compared to hard decisions. This might reflect that easier decisions could be based on unambiguous value representations, which could facilitate the decision process. This interpretation, however, remains to be tested in future studies.
(DOCX)
