# Supplementary material for: Led into Temptation? Rewarding Brand Logos Bias the Neural Encoding of Incidental Economic Decisions
Source: PLoS One. 2012 Mar 30;7(3):e34155. doi: 10.1371/journal.pone.0034155 (PMC3316633; doi:10.1371/journal.pone.0034155)
Supplement: Table S1 — Questionnaire responses. Note: Sbj = subject. Ratings from 1 (strongly disagree) to 7 (strongly agree). Salience = “When I think of electronic products such as MP3 players, mobile phones, or computers, this brand is one of the first brands that comes to mind: [Apple logo]”; Love = “I love this brand: [Apple logo]”; Own = “I own one or more products of this brand: [Apple logo]”; Desire = “I would like to own one or more products of this brand: [Apple logo]”; Intent = “I plan to buy one or more products of this brand in the next 6 months: [Apple logo]”; BIQ = sum of BIS-11 Attention, Motor, and Self-Control factor scores. (DOCX) [file pone.0034155.s001.docx]

Murawski, Harris, Bode, Domínguez D., and Egan: Led into temptation? Rewarding brand logos bias incidental economic decisions

**Table S1: Questionnaire responses**

| **Sbj** | **Salience** | **Love** | **Own** | **Desire** | **Intent** | **BIQ** |
| --- | --- | --- | --- | --- | --- | --- |
|  |  |  |  |  |  | **score** |
| 1 | 6 | 6 | 1 | 6 | 4 | 4.3 |
| 2 | 6 | 7 | 1 | 7 | 6 | 7.4 |
| 3 | 5 | 4 | 1 | 2 | 1 | 4.9 |
| 4 | 6 | 5 | 1 | 6 | 5 | 6.8 |
| 5 | 6 | 5 | 1 | 6 | 2 | 4.8 |
| 6 | 5 | 6 | 1 | 5 | 4 | 5.5 |
| 7 | 5 | 4 | 0 | 7 | 6 | 6.3 |
| 8 | 4 | 3 | 0 | 6 | 4 | 7.0 |
| 9 | 6 | 5 | 0 | 5 | 4 | 6.3 |
| 10 | 7 | 5 | 1 | 5 | 5 | 6.1 |
| 11 | 7 | 5 | 1 | 6 | 4 | 7.5 |
| 12 | 5 | 4 | 1 | 5 | 2 | 5.7 |
| 13 | 5 | 5 | 1 | 7 | 4 | 6.8 |
