# Supplementary material for: Led into Temptation? Rewarding Brand Logos Bias the Neural Encoding of Incidental Economic Decisions
Source: PLoS One. 2012 Mar 30;7(3):e34155. doi: 10.1371/journal.pone.0034155 (PMC3316633; doi:10.1371/journal.pone.0034155)
Supplement: Table S2 — Results of visibility tests. Note: The table shows d′ for the two visibility test as well as bootstrapped 95% confidence intervals of d′. In Test 1 (2AFC prime vs. control blank screens), a hit was defined as a ‘yes’ response in a trial in which a prime was displayed and a false alarm was defined as a ‘yes’ response in a trial in which no prime was displayed. In Test 2 (4AFC), a hit was defined as an ‘Apple’ response in a trial in which the Apple logo was displayed and a false alarm was defined as an ‘Apple’ response in a trial in which a different prime was displayed. Confidence intervals were bootstrapped at subject-level by resampling responses from the empirical distributions and computing d′ based on resampled responses (n = 1000). Two of the participants, marked # in the table above, did not have any hits in the Apple condition and d′ could not be computed. (DOCX) [file pone.0034155.s002.docx]

Murawski, Harris, Bode, Domínguez D., and Egan: Led into temptation? Rewarding brand logos bias incidental economic decisions

**Table S2: Results of visibility tests**

| **Participant** | **Test 1** | | | **Test 2** | | |
| --- | --- | --- | --- | --- | --- | --- |
|  | **d'** | **95% CI** | | **d'** | **95% CI** | |
| 1 | -0.357^*^ | -1.194 | 0.441 | -1.106 | -1.493 | -0.143 |
| 2 | 0.168^*^ | -0.684 | 1.054 | -0.333^*^ | -0.723 | 0.552 |
| 3 | 0.177^*^ | -0.692 | 0.967 | -0.333^*^ | -0.802 | 0.552 |
| 4 | -0.514^*^ | -1.478 | 0.341 | -0.220^*^ | -0.922 | 0.723 |
| 5 | -0.659^*^ | -1.581 | 0.240 | 0.171^*^ | -0.723 | 0.992 |
| 6^#^ | 0.000^*^ | -0.955 | 0.861 | - | - | - |
| 7^#^ | -1.049 | -2.222 | -0.167 | - | - | - |
| 8 | 0.387^*^ | -0.411 | 1.282 | -0.552^*^ | -0.992 | 0.552 |
| 9 | -0.263^*^ | -1.095 | 0.524 | -0.440^*^ | -1.310 | 0.440 |
| 10 | 0.000^*^ | -0.871 | 0.827 | -0.534^*^ | -1.310 | 0.436 |
| 11 | -0.524^*^ | -1.451 | 0.304 | -0.314^*^ | -1.106 | 0.552 |
| 12 | 0.084^*^ | -0.771 | 1.049 | -0.866^*^ | -1.310 | 0.220 |
| 13 | -0.684^*^ | -1.590 | 0.168 | -0.220^*^ | -0.992 | 0.552 |
| ^#^ No hits in Test 2. ^*^ Test performance not significantly different from chance. | | | | | | |
