# Supplementary material for: Led into Temptation? Rewarding Brand Logos Bias the Neural Encoding of Incidental Economic Decisions
Source: PLoS One. 2012 Mar 30;7(3):e34155. doi: 10.1371/journal.pone.0034155 (PMC3316633; doi:10.1371/journal.pone.0034155)
Supplement: Table S3 — Comparison of baseline model and priming model. (a) Parameter estimates. Note: Sbj = subject; k = discount rate; SE = standard error of parameter estimate; omega = reciprocal of the variance of logistic distribution; a = priming premium. For equations and details see Materials and Methods. (b) Model comparison. Note: Sbj = subject; B = baseline model (equation I); P = priming premium model (equation III); Δ = P – B; p(LR) = p-value of likelihood ratio test; AIC = Akaike Information Criterion; BIC = Bayesian Information Criterion. (DOCX) [file pone.0034155.s003.docx]

Murawski, Harris, Bode, Domínguez D., and Egan: Led into temptation? Rewarding brand logos bias incidental economic decisions

**Table S3: Comparison of baseline model and priming model**

**(a) Parameter estimates**

|  | **Baseline model (equation I)** | | | | **Priming premium model (equation III)** | | | | | |
| --- | --- | --- | --- | --- | --- | --- | --- | --- | --- | --- |
| **Sbj** | **k** | **SE** | **omega** | **SE** | **k** | **SE** | **omega** | **SE** | **a** | **SE** |
|  |  |  |  |  |  |  |  |  |  |  |
| 1 | 0.028 | 0.001 | 1.894 | 0.10 | 0.029 | 0.001 | 2.195 | 0.09 | -0.119 | 0.06 |
| 2 | 0.030 | 0.002 | 2.845 | 0.12 | 0.030 | 0.002 | 2.875 | 0.12 | -0.033 | 0.06 |
| 3 | 0.014 | 0.004 | 8.472 | 0.04 | 0.014 | 0.004 | 7.769 | 0.05 | 0.183 | 0.15 |
| 4 | 0.059 | 0.005 | 4.908 | 0.04 | 0.070 | 0.003 | 3.222 | 0.05 | -0.822 | 0.10 |
| 5 | 0.003 | 0.001 | 1.439 | 0.16 | 0.002 | 0.001 | 0.958 | 0.23 | 0.093 | 0.02 |
| 6 | 0.008 | 0.001 | 4.277 | 0.05 | 0.008 | 0.001 | 4.278 | 0.05 | 0.000 | 0.06 |
| 7 | 0.030 | 0.002 | 2.852 | 0.07 | 0.028 | 0.002 | 2.261 | 0.08 | 0.262 | 0.06 |
| 8 | 0.030 | 0.003 | 4.449 | 0.05 | 0.027 | 0.003 | 4.102 | 0.05 | 0.320 | 0.13 |
| 9 | 0.029 | 0.003 | 4.052 | 0.06 | 0.024 | 0.002 | 3.471 | 0.06 | 0.373 | 0.13 |
| 10 | 0.050 | 0.002 | 0.638 | 0.31 | 0.047 | 0.002 | 0.602 | 0.28 | 0.207 | 0.05 |
| 11 | 0.012 | 0.001 | 2.929 | 0.08 | 0.010 | 0.001 | 2.731 | 0.08 | 0.175 | 0.07 |
| 12 | 0.020 | 0.005 | 4.087 | 0.05 | 0.005 | 0.001 | 1.223 | 0.20 | 0.270 | 0.09 |
| 13 | 0.021 | 0.006 | 10.178 | 0.03 | 0.022 | 0.007 | 10.268 | 0.03 | -0.149 | 0.18 |
|  |  |  |  |  |  |  |  |  |  |  |
| *Min* | *0.003* |  | *0.638* |  | *0.002* |  | *0.602* |  | *-0.822* |  |
| *Mn* | *0.028* |  | *4.052* |  | *0.024* |  | *2.875* |  | *0.175* |  |
| *Max* | *0.059* |  | *10.178* |  | *0.070* |  | *10.268* |  | *0.373* |  |

**(b) Model comparison**

|  | **Log Likelihood** | | | | **AIC** | | | **BIC** | | |
| --- | --- | --- | --- | --- | --- | --- | --- | --- | --- | --- |
| **Sbj** | **B** | **P** | **Δ** | **p(LR)** | **B** | **P** | **Δ** | **B** | **P** | **Δ** |
| 1 | -74.6 | -71.7 | 2.9 | 0.016 | 153.2 | 149.3 | -3.8 | 153.8 | 150.3 | -3.5 |
| 2 | -109.9 | -109.7 | 0.2 | 0.496 | 223.8 | 225.3 | 1.5 | 224.4 | 226.3 | 1.9 |
| 3 | -114.7 | -113.7 | 1.1 | 0.141 | 233.5 | 233.3 | -0.2 | 234.2 | 234.3 | 0.2 |
| 4 | -98.7 | -69.7 | 29.0 | 0.000 | 201.5 | 145.5 | -56.0 | 202.2 | 146.5 | -55.7 |
| 5 | -73.0 | -62.0 | 11.0 | 0.000 | 150.0 | 130.1 | -20.0 | 150.7 | 131.1 | -19.6 |
| 6 | -81.8 | -81.8 | 0.0 | 1.000 | 167.6 | 169.6 | 2.0 | 168.3 | 170.6 | 2.3 |
| 7 | -84.8 | -72.9 | 11.9 | 0.000 | 173.5 | 151.7 | -21.8 | 174.2 | 152.7 | -21.5 |
| 8 | -95.6 | -90.0 | 5.6 | 0.001 | 195.2 | 186.0 | -9.2 | 195.9 | 187.0 | -8.9 |
| 9 | -92.0 | -82.7 | 9.3 | 0.000 | 187.9 | 171.4 | -16.5 | 188.6 | 172.4 | -16.2 |
| 10 | -34.5 | -19.3 | 15.1 | 0.000 | 73.0 | 44.7 | -28.3 | 73.7 | 45.7 | -28.0 |
| 11 | -89.4 | -84.3 | 5.1 | 0.001 | 182.9 | 174.6 | -8.3 | 183.6 | 175.6 | -7.9 |
| 12 | -77.8 | -51.8 | 26.0 | 0.000 | 159.6 | 109.6 | -50.1 | 160.3 | 110.6 | -49.7 |
| 13 | -120.4 | -119.9 | 0.5 | 0.307 | 244.8 | 245.8 | 1.0 | 245.5 | 246.8 | 1.3 |
