# Supplementary material for: Led into Temptation? Rewarding Brand Logos Bias the Neural Encoding of Incidental Economic Decisions
Source: PLoS One. 2012 Mar 30;7(3):e34155. doi: 10.1371/journal.pone.0034155 (PMC3316633; doi:10.1371/journal.pone.0034155)
Supplement: Table S4 — Mixed-effects model of priming effect. Note: The table above shows estimates of fixed effects in a non-linear mixed effects model of participants' choices at group level based on Equation (III). Standard errors of coefficient estimates are given in brackets. *** p<0.001; ** p<0.01; * p<0.05. (DOCX) [file pone.0034155.s004.docx]

Murawski, Harris, Bode, Domínguez D., and Egan: Led into temptation? Rewarding brand logos bias incidental economic decisions

**Table S4: Mixed-effects model of priming effect**

|  | **Priming premium model (III)** | | |
| --- | --- | --- | --- |
| k |  | **0.023^***^** (0.004) |  |
| a |  | **0.063^***^** (0.004) |  |
| omega |  | **0.715^***^** (0.152) |  |
|  |  |  |  |
| N |  | 2,800 |  |
| LL |  | -1,098 |  |
| AIC |  | 2,209 |  |
