# Supplementary material for: Led into Temptation? Rewarding Brand Logos Bias the Neural Encoding of Incidental Economic Decisions
Source: PLoS One. 2012 Mar 30;7(3):e34155. doi: 10.1371/journal.pone.0034155 (PMC3316633; doi:10.1371/journal.pone.0034155)
Supplement: Table S5 — Baseline contrasts for univariate analyses. Notes: Temp. Disc. = temporal discounting decisions; Control = perceptual control decisions; SV param = parametric effect for general subjective value (no priming effect accounted for); L = left; R = right; clusters determined by Z value>1.96 and a family-wise error (FWE) corrected cluster significance threshold of p<0.05; coordinates are given in MNI space. Please note that the perceptual control task also activated parts of the same network as the temporal discounting task and thus did not constitute an optimal task to isolate regions that were uniquely involved in discounting. (DOCX) [file pone.0034155.s005.docx]

Murawski, Harris, Bode, Domínguez D., and Egan: Led into temptation? Rewarding brand logos bias incidental economic decisions

**Table S5: Baseline contrasts for univariate analyses**

| **Anatomical area** | **L/R** | ***Z* max** | **x** | **y** | **z** |
| --- | --- | --- | --- | --- | --- |
| **TEMP. DISC. > BASELINE** |  |  |  |  |  |
| visual cortex | L | 6.33 | -26 | -88 | -10 |
|  | R | 6.06 | 32 | -78 | -14 |
| insula | R | 5.18 | 36 | 16 | 0 |
|  | L | 4.59 | -34 | 18 | 2 |
| intraparietal sulcus | R | 4.90 | 40 | -56 | 46 |
|  | L | 4.82 | -28 | -56 | 40 |
| temporal pole | R | 4.85 | 48 | 16 | -8 |
| dorsolateral prefrontal cortex | L | 4.66 | -36 | 50 | 8 |
|  | R | 3.66 | 38 | 40 | 8 |
| anterior cingulate cortex | L | 4.58 | -6 | 33 | 20 |
|  | R | 4.15 | 4 | 32 | 6 |
| ventral striatum | R | 4.72 | 26 | 16 | 4 |
|  | L | 3.87 | -24 | 2 | -10 |
|  |  |  |  |  |  |
| **SV PARAM. > BASELINE** |  |  |  |  |  |
|  |  |  |  |  |  |
| medial prefrontal cortex | R | 2.73 | 6 | 40 | 24 |
| dorsolateral prefrontal cortex | R | 2.97 | 46 | 10 | 54 |
| caudate nucleus | L | 3.04 | -6 | 12 | -2 |
|  | R | 3.27 | 12 | 12 | 12 |
| nucleus accumbens | R | 2.53 | 10 | 6 | -2 |
| thalamus | R | 2.82 | 4 | 0 | 0 |
|  |  |  |  |  |  |
| **TEMP. DISC. > CONTROL** |  |  |  |  |  |
| visual cortex | R | 4.33 | 26 | -94 | -4 |
|  | L | 3.94 | -32 | -92 | -8 |
| anterior cingulate cortex | R | 3.62 | 8 | 20 | 42 |
|  | L | 3.39 | -8 | 16 | 44 |
| inferior prefrontal cortex | R | 3.55 | 48 | 8 | 22 |
|  | L | 3.46 | -38 | 8 | 26 |
| intraparietal sulcus | R | 4.29 | 30 | -60 | 40 |
| midbrain |  | 3.69 | -2 | -24 | -16 |
| precentral gyrus | L | 3.71 | -38 | -4 | 42 |
|  | R | 3.19 | 38 | -10 | 38 |
| insula | L | 3.39 | -40 | 14 | 12 |
|  | R | 3.11 | 34 | 22 | 2 |
