# Supplementary material for: Led into Temptation? Rewarding Brand Logos Bias the Neural Encoding of Incidental Economic Decisions
Source: PLoS One. 2012 Mar 30;7(3):e34155. doi: 10.1371/journal.pone.0034155 (PMC3316633; doi:10.1371/journal.pone.0034155)
Supplement: Table S6 — Results decoding decision aspects. Note: TD = temporal discounting decisions; L = left; R = right; M = mean; SEM = standard error of mean; apple = Apple prime condition; cup = neutral cup prime condition; mPFC = medial prefrontal cortex; ant = anterior; inf = inferior; DLPFC = dorsolateral prefrontal cortex; FPC = frontopolar cortex; temp = temporal; sulc = sulcus; gyr = gyrus; ‘#’ 2 subjects excluded because of unbalanced runs for both prime conditions (n = 11); all results p<0.05 family-wise error (FWE) corrected at cluster level; all analyses performed using unbiased searchlight decoding with radius r = 3 voxels. Coordinates are given for peak voxel in MNI space. (DOCX) [file pone.0034155.s006.docx]

Murawski, Harris, Bode, Domínguez D., and Egan: Led into temptation? Rewarding brand logos bias incidental economic decisions

**Table S6: Results decoding decision aspects**

| **Decoding** | **Anatomical area** | **L/R** | **Accuracy [%]** | | ***t*** | **x** | **y** | **z** |
| --- | --- | --- | --- | --- | --- | --- | --- | --- |
|  |  |  | **M** | **SEM** |  |  |  |  |
|  | | | | | | | | |
| **DECODING PRIME CONDITION** | | | | | | | | |
| apple vs cup | visual cortex | L | 58 | 0.32 | 4.32 | -16 | -99 | 8 |
|  | mPFC | L | 56 | 0.44 | 4.31 | -20 | 40 | 12 |
|  | | | | | | | | |
| **DECODING DECISION OUTCOME TD (NOW VS. LATER)** | | | | | | | | |
| TD (all) | orbito-frontal | R | 58 | 0.92 | 3.91 | 36 | 36 | 12 |
|  | | | | | | | | |
| **DECODING DECISION DIFFICULTY (EASY VS. HARD)** | | | | | | | | |
| TD (all) | ant mPFC | L | 61 | 0.79 | 5.09 | -8 | 60 | 8 |
| apple (#) | ant mPFC | L | 58 | 0.82 | 5.17 | -12 | 60 | 8 |
|  | postcentral sulc | L | 60 | 0.81 | 3.85 | -24 | -36 | 48 |
|  | inf temp gyr | L | 52 | 0.02 | 3.86 | 52 | -72 | -20 |
| cup (#) | ant mPFC | L | 54 | 0.75 | 4.07 | -12 | 60 | 8 |
|  | mPFC / FPC | R | 59 | 1.24 | 4.08 | 8 | 56 | 32 |
|  | postcentral sulc | L | 58 | 0.92 | 4.29 | -20 | -40 | 68 |
|  | inf temp gyr | L | 57 | 1.33 | 3.55 | -48 | -60 | -12 |
