# Supplementary material for: Led into Temptation? Rewarding Brand Logos Bias the Neural Encoding of Incidental Economic Decisions
Source: PLoS One. 2012 Mar 30;7(3):e34155. doi: 10.1371/journal.pone.0034155 (PMC3316633; doi:10.1371/journal.pone.0034155)
Supplement: Table S7 — Differential contrasts univariate analyses. Note: TD = temporal discounting decisions; Cup = neutral cup prime condition; L = left; R = right; clusters determined by Z value>1.96 and a family-wise error (FWE) corrected cluster significance threshold of p<0.05; coordinates are given in MNI space. Other contrasts (hard>easy; cup>apple; apple>cup; apple-hard>apple-easy; apple-easy>apple-hard; cup-hard>cup-easy) did not reveal significant results. Interestingly, easy choices led to more activation in anterior mPFC compared to hard decisions. This might reflect that easier decisions could be based on unambiguous value representations, which could facilitate the decision process. This interpretation, however, remains to be tested in future studies. (DOCX) [file pone.0034155.s007.docx]

Murawski, Harris, Bode, Domínguez D., and Egan: Led into temptation? Rewarding brand logos bias incidental economic decisions

**Table S7: Differential contrasts univariate analyses**

| **Anatomical area** | **L/R** | ***Z* max** | **x** | **y** | **z** |
| --- | --- | --- | --- | --- | --- |
|  |  |  |  |  |  |
| **EASY > HARD (all TD)** |  |  |  |  |  |
|  |  |  |  |  |  |
| superior frontal gyrus | L | 3.31 | -16 | 24 | 56 |
|  | R | 3.19 | 24 | 44 | 46 |
| frontopolar cortex (FPC) | R | 3.27 | 22 | 62 | 10 |
| anterior cingulate cortex (ACC) | R | 2.94 | 6 | 40 | 24 |
| medial prefrontal cortex (mPFC) | R | 2.91 | 6 | -58 | -6 |
|  |  |  |  |  |  |
| **CUP-EASY >CUP-HARD** |  |  |  |  |  |
|  |  |  |  |  |  |
| anterior mPFC / FPC | R | 3.25 | 4 | 42 | 24 |
